# An active inference account of stuttering behavior

**DOI:** 10.3389/fnhum.2025.1498423

**Published:** 2025-04-03

**Authors:** Evan Usler

**Affiliations:** Department of Communication Sciences and Disorders, University of Delaware, Newark, DE, United States

**Keywords:** stuttering, active inference, fluency, speech production, disfluency

## Abstract

This paper presents an interpretation of stuttering behavior, based on the principles of the active inference framework. Stuttering is a neurodevelopmental disorder characterized by speech disfluencies such as repetitions, prolongations, and blocks. The principles of active inference, a theory of predictive processing and sentient behavior, can be used to conceptualize stuttering as a disruption in perception-action cycling underlying speech production. The theory proposed here posits that stuttering arises from aberrant sensory precision and prediction error dynamics, inhibiting syllable initiation. Relevant to this theory, two hypothesized mechanisms are proposed: (1) a mistiming in precision dynamics, and (2) excessive attentional focus. Both highlight the role of neural oscillations, prediction error, and hierarchical integration in speech production. This framework also explains the contextual variability of stuttering behaviors, including adaptation effects and fluency-inducing conditions. Reframing stuttering as a synaptopathy integrates neurobiological, psychological, and behavioral dimensions, suggesting disruptions in precision-weighting mediated by neuromodulatory systems. This active inference perspective provides a unified account of stuttering and sets the stage for innovative research and therapeutic approaches.

## 1 Reframing stuttering: a predictive perspective

Stuttering is a complex fluency disorder that is often neurodevelopmental (i.e., childhood-onset fluency disorder) and sometimes acquired due to illness or injury. For most individuals, intricate coordination of the speech subsystems involved in respiration, articulation, and phonation results in a smooth and efficient flow of speech. For people who stutter (PWS), agentic control over speech is frequently compromised by stuttering behavior or ‘disfluency,’ traditionally classified as blocks, prolongations, or repetitions (Bloodstein et al., 2021). Beyond this core stuttering behavior, more peripheral behavior includes overt signs of tension and struggle in communication, circumlocution, and avoidance of feared words and social situations. Many PWS experience covert feelings such as self-consciousness, shame, and apprehension. These emotions often contribute to a perceived loss of control over their communication (Bloodstein et al., 2021; Perkins, 1990). This multifaceted experience highlights the need for comprehensive models to explain the variability of stuttering behavior.

Recent accounts of stuttering behavior highlight neurodevelopmental (Smith and Weber, 2017), genetic (Kraft and Yairi, 2012), biological (Neef and Chang, 2024), and computational (Chang and Guenther, 2019) factors, shedding light on the multifaceted nature of stuttering. However, some aspects of stuttering behavior appear to be functional or psychogenic (Utianski and Duffy, 2022; Bloodstein, 1949). In a Presidential town hall in February 2020, President Joe Biden openly pondered the cause of stuttering and how he could overcome its debilitating effects (CNN, 2020). He explained, “*I think part of it is confidence and how you were [sic] what circumstance you face…*” This folk psychological view of stuttering, which assumes an internal locus of control and role of self-confidence in stuttering behavior, remains dominant in everyday life. The current zeitgeist around stuttering includes conflicting narratives regarding the contextual nature and (in)tractability of stuttering behavior. Despite over a century of empirical research, it remains unclear why stuttering occurs during some communicative situations and not in others, and what role confidence is likely to play in this variability. However, in the past two decades, the application of Bayesian mechanics to the cognitive-and neuro-sciences has advanced probabilistic frameworks for a greater understanding of the brain and behavior in contexts of varying confidence and uncertainty (Doya et al., 2011; Clark, 2015).

An increasingly influential theory of sentient behavior known as *active inference* provides a novel explanation of stuttering as a pathology of aberrant predictive processing (Pezzulo et al., 2024). This framework describes how biological agents predict and adapt to their environment (Pezzulo et al., 2024; Parr and Pezzulo, 2022), including both maintenance of optimal internal states (i.e., homeostasis) and adaptability during times of stress (i.e., allostasis) (Seth and Friston, 1708; Barrett et al., 2016; Schulkin and Sterling, 2019). Active inference provides an explanation of stuttering behavior as an involuntary, transient, and chronic failure by the speaker to self-attenuate from the act of speaking. More specifically, atypical precision (i.e., confidence) afforded to perception-action cycling may abruptly inhibit syllable initiation, resulting in stuttering behavior. This theory links neurobiological and psychological views of stuttering by framing stuttering as a synaptopathy – a disorder in the precision afforded to parameters of predictive processing mediated by various neuromodulatory systems.

## 2 Core concepts of active inference

According to this framework, based on the free energy principle, biological agents have an existential imperative to minimize uncertainty (or, more technically, variational free energy) by generating internal probabilistic representations of their environment (Friston et al., 2022). The brain operates as a Bayesian inference organ that infers the probable causes of sensory input from the environment via predictive coding (Rao and Ballard, 1999). A technical account of active inference and the free energy principle is beyond the scope of this article but is available elsewhere (Pezzulo et al., 2024; Friston et al., 2022). The brain operates as a hierarchical generative model comprising of prior expectations *[P(x)]*, representing the brain’s existing beliefs or predictions about certain states or events. Additionally, it includes likelihood functions *[P(y|x)]*, which quantify the probability of receiving specific sensory inputs *(y)* given a particular state of the world *(x)*. To clarify, the likelihood function plays a crucial role in how the brain interprets incoming sensory information by assessing how probable these sensations are if a certain hypothesis about the environment is true. For instance, when you expect to hear and feel your speech (i.e., prior expectation) and then actually receive the feedback (i.e., sensory input), the likelihood function evaluates how well the sensory input aligns with your expectation. This process is closely tied to sensorimotor integration, where sensory input and motor actions are seamlessly combined to form coherent perceptions and drive behavior. Sensorimotor integration ensures that the brain’s predictions are continuously updated based on real-time sensory feedback. When there’s a mismatch between expectations and sensory input, the likelihood function helps adjust the posterior expectations based on the precision of prediction errors, refining the brain’s model of the environment for more accurate future predictions. By integrating prior expectations with likelihood functions through sensorimotor interactions, the brain maintains a dynamic and adaptive understanding of the world, enabling efficient processing in response to changing conditions.

The interplay between Bayesian priors, likelihoods, and posteriors ensures that speech-language production is a context-sensitive and dynamic process in which agents continuously generate syllables, words, and phrases while adjusting to situation-specific prior knowledge and sensory feedback. Bayesian inference provides a useful framework for understanding how this process balances accuracy in model predictions and flexibility in model updating. For example, prior expectations include the words likely to be used in the current situation. The likelihood reflects how well each potential word fits the specific context. Prior expectations are integrated with the likelihood of arriving at the most probable and relevant word (i.e., posterior). However, sensory feedback from oneself or perhaps a communicative partner, can refine this likelihood. This fosters speech-language production that is both efficient and adaptive.

Simply put, perception is conceptualized as a form of predictive processing in which prediction error is minimized by Bayesian model updating. Descending predictions minimize ascending prediction errors and thus update the model. The higher up the model hierarchy, the slower and more generalized predictions and prediction errors become. Over time, prior expectations become increasingly veridical to the environment while still being open to new sensory input from a dynamic world. While prediction error minimization during predictive processing depends on past experiences and current sensory input, complex agents also minimize *expected* prediction error by planning sequences of action into the future (Friston, 2010). An agent selects an optimal sequence of action or *policy* that is predicted to result in the proprioceptive, exteroceptive, and interoceptive endpoints of a future desired state.

Highly precise prior expectations reflect high confidence or certainty. On the contrary, uncertainty and anxiety are similarly conceptualized as the inability to reliably minimize prediction error through perception and action (Peters et al., 2017; Pezzulo and Friston, 2019). Prediction errors that are frequent, large, or unresolved signal to higher levels of the generative model that it is inadequate in predicting the environment. In active inference, (un)certainty refers to a quantitative measure of precision (or inverse variance) of a probability distribution representing a Bayesian parameter, such as prior expectations and sensory input (Yon and Frith, 2021). Precision is a second-order prediction of ‘context’ (e.g., how well you hear an utterance) associated with a prediction of ‘content’ (e.g., what utterance you expect to hear). Precision determines how much weight the agent gives to sensory data versus prior expectations when updating its generative model. ‘Prior precision’ is the confidence of our prior expectations from previous knowledge and experience. ‘Sensory precision’ is the confidence in the fidelity (i.e., likelihood) of the sensory input. Higher sensory precision implies greater confidence in the sensory input and, as a result, the agent relies more on sensory input for updating expectations. Conversely, low sensory precision results in a greater reliance on prior expectations. Thus, when prior precision is strong, sensory precision is relatively weak, and vice versa. Precision weighting can be viewed as a form of gain control to the influence of sensory input on updating the generative model. In other words, prediction error is minimized by perception (i.e., changing our model) when sensory precision is relatively strong, or by action (i.e., changing our environment) when sensory precision is relatively weak. Framing the brain and behavior as existentially minimizing prediction error via precision dynamics and the integration of perception and action can provide a new and nuanced view of speech fluency and stuttering behaviors.

## 3 The predictive path to speech fluency

The nature and clinical importance of ‘fluency’ has sparked enduring debate within the communication sciences (Tichenor et al., 2022). Traditionally, speech fluency has been conceptualized as the transitional smoothness or efficiency of perceptual features (e.g., acoustics, kinematics), and the absence of discrete interruption (e.g., disfluency). However, recent attempts to broaden the concept of speech fluency have emphasized the efficiency of achieving communicative goals and values (Usler, 2022a). In the context of active inference, fluency can be defined as the subjective experience of efficiency in cognitive, linguistic, and motor processes—a metacognitive signal indicating prediction error is reliably minimized via the successful integration of prior expectations and sensory input (Brouillet and Friston, 2023).

**Figure 1:**
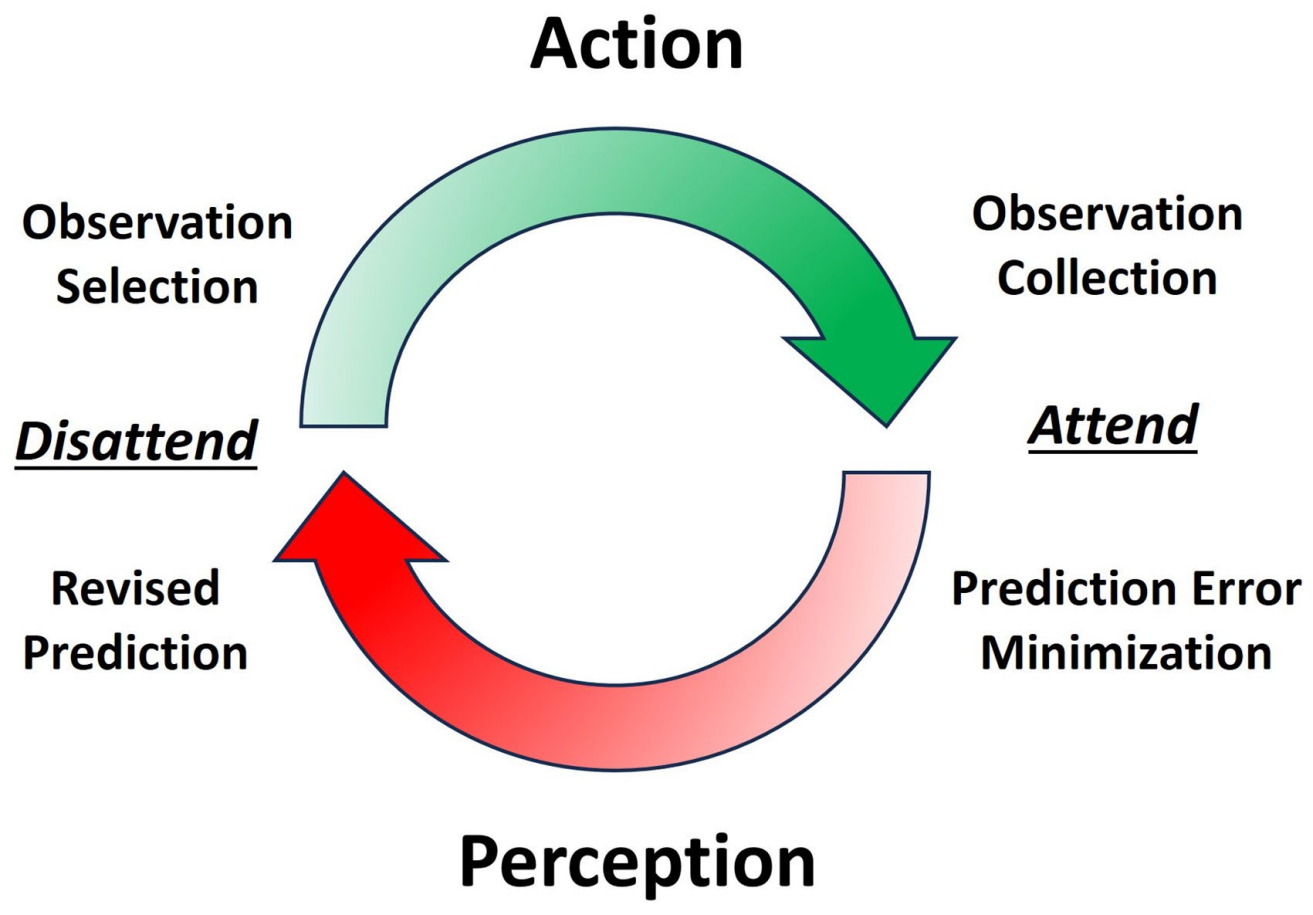
Perception-action cycle is the transition between collecting sensory observations through action and updating the generative model through perception. Attending facilitates perception by increasing sensory precision while disattending facilitates action by decreasing sensory precision.

Speech production is driven by perception-action cycles, influenced by the balance of prior and sensory precisions (See Figure 1) (Anil Meera et al., 2022). Sensory precision increases relative to prior precision with the prioritization of salient observations, commonly known as attention (Feldman and Friston, 2010). During speech, the speaker receives different sources of simultaneous sensory input: exteroceptive (e.g., auditory), proprioceptive (e.g., somatosensory), and interoceptive (e.g., autonomic). These sensory inputs inform the speaker of progress in communicating and, relatedly, how precision should be balanced between prior expectations and sensory input. This minimization process is analogous to the scientific method of prediction-making and observation collection (Anil Meera et al., 2022). Sequential perception-action cycles underlying behavior, such as saccades (Parr et al., 2019; Hoffman et al., 2013) and syllables (Poeppel and Assaneo, 2020; Pellegrino et al., 2011), are largely produced in a theta frequency (3–8 Hz) (Fiebelkorn and Kastner, 2019; Benedetto et al., 2020). The syllable is the likely unit of information processing for speech perception and production (e.g., ‘theta-syllable’) (Ghitza, 2013; Strauß and Schwartz, 2017). For our purposes, the syllable is viewed as a computationally-and biomechanically constrained temporal scheduling of perception-action cycles that underlie fluency in speech-language production.

Understanding the mechanisms behind speech fluency requires examining the interaction between sensory input and motor output in a dynamic context. Active inference is a revised ideomotor theory in which action is not driven by descending motor commands but by ascending prediction errors (Adams et al., 2013; Mohan et al., 2019). An action is performed to minimize prediction error between a precise prior expectation of the sensory input (associated with a future goal state) and current sensory input (evidencing the goal state has not yet been attained). To do so, one must reduce sensory precision relative to prior precision (i.e., sensory attenuation) by disattending to current sensory input (Brown et al., 2013). Sensory attenuation is a reduction of sensation intensity (Blakemore et al., 2000), which is required for action initiation according to the ideomotor principles of active inference. Put simply, the brain ‘fools itself’ into movement by not considering the current sensory evidence that movement has yet to occur. Instead, strong prior expectations of sensory endpoints (that have yet to be reached) result in prediction error that is only resolved by the movement. This notion of movement as a self-fulfilling prophecy requires sensory attenuation and action initiation to occur in synchrony for optimal perception-action cycling. A speaker must effortlessly modulate their prior and sensory precisions with just the right timing and rhythm to produce the syllable sequencing that we perceive as fluent speech. It follows that a failure to attenuate sensory precision at syllable onset disrupts forward action, as there is no prediction error to minimize.

Although recent accounts of motor control based on active inference principles have focused on proprioceptive prediction error (Parr et al., 2021), speech production is also refined by simultaneous exteroceptive (i.e., auditory) feedback. As stated by Najnin and Banerjee, “instead of directly mapping from auditory sensory to action, auditory and proprioceptive sensory can be fused and action can be inferred from proprioceptive sensory prediction error” (Najnin and Banerjee, 2017). A detailed active inference model of speech perception and production is beyond the scope of this article, but recent conceptualizations are available elsewhere (Najnin and Banerjee, 2017; Friston et al., 2021; Bradshaw et al., 2024). Instead of viewing stuttering as being caused by a disorder of speech motor planning, execution, or coordination, stuttering may be viewed as a disorder of inference. Stuttering behavior may dynamically emerge when sequential perception-action cycles fail to transition accordingly, leading to transient moments of stuckness and a loss of control over speech-language production.

This ideomotor approach contrasts with contemporary speech production models that emphasize articulatory kinematics and inverse modeling (Parrell et al., 2019), including two models that have influenced current perspectives on stuttering: the Directions Into Velocities of Articulators (DIVA) (Tourville and Guenther, 2011) and the Hierarchical State Feedback Control (HSFC) models (Hickok et al., 2011). According to the DIVA model, speech production is driven by stored feedforward motor commands refined by errors with predictive somatosensory and auditory targets. From this perspective, unstable internal models (Max et al., 2004), atypical dopaminergic signaling (Civier et al., 2013), and aberrantly high reliance on sensory feedback (Civier et al., 2010) associated within the basal ganglia-thalamo-cortical loop result in stutter-like disruptions in simulated speech. In the HSFC model, speech production is driven by an internal model of the vocal tract that supports internal monitoring to determine the accuracy of current motor commands relative to intended sensory targets. Stuttering behaviors are thus triggered by error correction of inaccurate motor predictions due to ‘noisy’ sensorimotor integration (Hickok et al., 2011). Contemporary models of speech-language production, such as the DIVA and HSFC models, emphasize specific neural mappings to specific linguistic and motoric processes, and are heavily influenced by control theory that posits that movement is driven by the optimization of a value function towards a desired endpoint.

**Figure 2:**
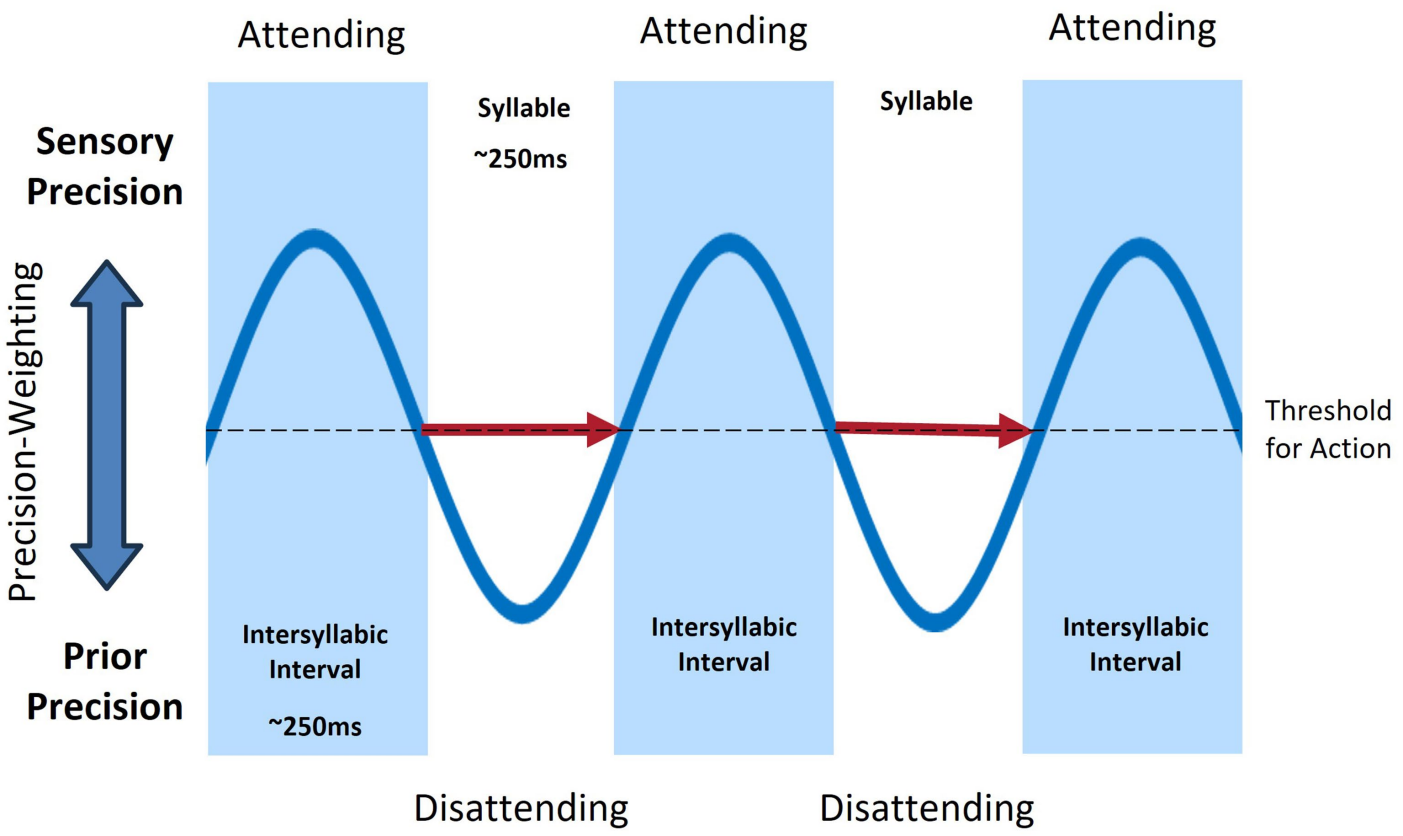
Rhythmic fluctuations of sensory and prior precisions (blue), associated with attention, during sequential syllable production (red arrows).

On the contrary, in the active inference framework, motor control is a form of Bayesian inference to minimize prediction error (Friston, 2011). All neural processes are Bayesian with predictions and prediction errors framed as probability distribution with an associated precision (i.e., inverse variance). All environmental input is simply sensory prediction error and the precision of that input (whether or not it is likely to influence higher-levels of the generative model) is mediated by attentional mechanisms. In active inference, an inverse model for the formulation of learned motor representations that are then executed via a feedforward mechanism is not required. Consistent with the equilibrium-point hypothesis (Feldman, 2023; Perrier et al., 1996), movements are not centrally planned and executed but arise when prior expectations about sensations differ from current sensory input (Adams et al., 2013). This view of motor control closely aligns with the activation of mirror neurons during movement perception and performance (Rizzolatti et al., 1996). Thus, movement likely occurs through the sequencing of perception-action cycles, modulated by fluctuations in sensory precision, and without the need for distinct forward and inverse modeling (See Figure 2). Prediction errors are minimized at the lowest level of the hierarchical model by closed-loop motor reflex arcs in the brainstem and spinal cord that bring the position of relevant effectors into line with predicted proprioceptive endpoints.

Models of speech production that rely on control theory versus active inference principles offer complementary perspectives on why stuttering behaviors occur. From a control theory perspective, speech production relies on a balance between feedforward and feedback control—fluent speech relies on strong feedforward processes and minimal reliance on feedback, particularly in highly automatized speech. This is analogous to active inference, in which strong prior precision and weak sensory precision drive well-learned (i.e., automatic) or highly predictable speech. However, active inference conceptualizes speech-language production as a hierarchical Bayesian process, explicitly incorporating uncertainty via precision-weighting. This is critical for understanding the dynamic and contextual nature of stuttering behavior.

## 4 The role of uncertainty in stuttering behavior

To investigate the proximate cause(s) of stuttering behavior, it is first reasonable to look at the communicative contexts in which stuttering occurs. Developmental stuttering often begins when children start producing multisyllabic utterances, highlighting the importance of syllable sequencing (Bloodstein et al., 2021). Stuttering behavior typically occurs when utterances, such as words and sentences, are initiated (Brown, 1945; Buhr and Zebrowski, 2009) and follow a “declining gradient of incidence” (Quarrington, 1965). Stuttering also tends to be elicited by increased word unpredictability or information content (Quarrington, 1965; Schlesinger et al., 1965), utterance length (Lanyon and Duprez, 1970; Soderberg, 1971), and contextual relevance (Kaasin and Bjerkan, 1982; Byrd et al., 2011). Notably, words deemed ‘critical’ to the overall message are often stuttered (Kaasin and Bjerkan, 1982; Byrd et al., 2011; Eisenson and Horowitz, 1945; Eisenson and Wells, 1942). These different variables, which similarly elicit stuttering in both developmental and acquired forms of the disorder (Max et al., 2019), appear to be additive in their tendency to elicit stuttering (Schlesinger et al., 1965) while also eliciting stuttering independently (Quarrington, 1965). As Taylor (1966) argued, the influence of all these different variables on the variability of stuttering can be explained by the metavariable of uncertainty. By framing the various situational factors that elicit stuttering—such as word unpredictability, utterance length, and contextual relevance—under the broader metavariable of uncertainty, we can better understand how these elements collectively influence speech production and its disruption, as well as the potential role of confidence in modulating these effects.

In active inference, a failure to accurately and reliably predict sensory input from an ever-changing environment has been associated with the psychological states of anxiety, uncertainty, and hypervigilance (Peters et al., 2017). Epistemic uncertainty is a lack of information about how to act in a communicative situation. This uncertainty is resolved by the information gain from sensory input, which ‘self-evidences’ the speaker as an effective communicator (Hohwy, 2022). In other words, a speaker’s own speech reduces uncertainty regarding what, when, and how particular syllable sequences could be produced next. Another form of uncertainty is ‘volatility’—the intrinsic unpredictability of the communicative landscape that the environment affords (Dayan and Yu, 2002; Vincent et al., 2019). For instance, reciting a remembered poem to a favorite pet entails little volatility. Conversely, spontaneously answering questions at a job interview presents high volatility. Policies, or sequences of action, require transitions from one state (e.g., syllable) to the next, and this ‘transition precision’ is the inverse of volatility (Parr and Friston, 2017). Furthermore, policies also have a ‘policy precision’ relative to competing policies for initiation. To best minimize expected prediction error, highly precise policies are chosen because they are optimally accurate yet as simple as possible (Friston et al., 2022). Syllable sequences with high policy and transition precisions, such as commonly used phrases or sentences, allow the speaker to ‘know exactly what to say and how to say it’ and thus foster a sense of agentic control over communication.

The notion that uncertainty plays a role of cause (Taylor, 1966) and effect (Boyle and Chagachbanian, 2022) on stuttering has been long supposed. However, the mechanism that mediates the relationship between uncertainty and stuttering has remained elusive. Behavioral and neurophysiological evidence support the claim that PWS exhibit imprecise prior expectations, compared to non-stuttering peers, regarding the sensory endpoints of their motor output. For example, adults who stutter (AWS) are relatively less accurate in predicting the consequences of upper limb movement (Daliri et al., 2014). Greater variability in articulatory coordination has been repeatedly associated with stuttering in children and adults (Usler et al., 2017; Smith et al., 2010). PWS also exhibit relative limitations in speech motor skills (Namasivayam and van Lieshout, 2011; Van Lieshout et al., 2004) and the speech of PWS is less rhythmic than that of non-stuttering peers (Boecher et al., 2025). Children who stutter (CWS), on average, exhibit maturational lags in their speech-language development (Smith and Weber, 2017), and numerous studies have associated stuttering with atypical event-related potential (ERP) amplitudes to auditory and linguistic prediction errors (Gastaldon et al., 2023; Maxfield et al., 2010; Weber-Fox, 2001). From an active inference perspective, ERP amplitudes index the precision-weighting of prediction errors ascending the model hierarchy (Smith et al., 2022). Although ERP findings have varied [for a review, see Bloodstein et al. (2021)], PWS often exhibit generally reduced ERP amplitudes compared to non-stuttering peers, suggesting less precise prior expectations facilitating their speech-language predictions. Recognizing how uncertainty shapes stuttering behavior provides a pathway to more profound insights into the role of precision dynamics underlying Bayesian inference.

## 5 Precision dynamics and stuttering behavior

How does uncertainty elicit stuttering behavior? Weak prior expectations regarding the likely causes of incoming sensations increase the relative precision afforded to the sensory input via selective attention. For example, if you hike a well-trodden path, high confidence in the appropriate sequence of actions allows you to disattend from the sensory environment. However, if your prior expectations result in prediction error and uncertainty (e.g., you realize you are suddenly lost), attention boosts precision to the most informative sensory stimuli within the environment. As long as volatility is low (e.g., it is a clear and sunny day), epistemic uncertainty is likely to be reduced by adaptive actions that boost information gain (e.g., looking for the helpful trail sign). Thus, boosting sensory precision during times of stress is an adaptive way to increase the rate of model updating (e.g., realizing where you had gone off trail) and adapting behavior accordingly. Speech-language production can be similarly viewed as sequential perception-action cycles for the resolution of prediction error. Analogous to our hypothetical hiking example is the experience of a child with maturing linguistic, cognitive, and speech-motor abilities now traversing the increasingly complex linguistic landscape during the early preschool years—a critical period in which stuttering is likely to emerge, as well as disappear (Smith and Weber, 2017). Unlike language and articulation disorders, stuttering typically emerges after an initial period of speech-language development (Bloodstein et al., 2021). Stuttering behaviors may suddenly occur when children are beginning to sequence syllables and words according to syntactic rules. Although stuttering in adults largely occurs on highly informative content words, early stuttering often occurs on function words. The immature syntactic abilities of children reduce the predictability of function words, thus boosting sensory precision. One can speculate that a minority of children, during a rapid period of language development, may fail to develop a tendency to disattend to their own speech, particularly at the location of challenging language formulation, such as function words and the beginning of syntactic structures. After years of linguistic practice, the predictable nature of function words, facilitated by their high rate of occurrence and lack of information content, likely reduces sensory precision afforded to their onset. On the contrary, stuttering is likely to continue on those content words that are less frequently spoken and hold high information content.

Environmental conditions that increase uncertainty in task performance also increase sensory precision via selective attention for the minimization of that uncertainty. It follows that during periods of uncertainty in the sensory consequences of one’s speech production, perhaps due to weak prior expectations or volatility in the communicative environment, stuttering behaviors could be elicited by a lack of sensory attenuation that suddenly inhibits syllable initiation. From an ideomotor perspective, there is simply no prediction error for action to resolve because current sensory input (that speech had yet to occur) is more precise than that of the expected sensory consequences of movement. This increased sensory precision thus prevents the sensory attenuation required for the initiation of action. Such phenomena would explain why stuttering predominantly occurs at the beginning of words and phrases (Quarrington, 1965). Sensory attenuation is evidenced by a decrease in ERP amplitude to self-generated versus external stimuli (Mifsud and Whitford, 2017; Timm et al., 2016; Saupe et al., 2013; Horváth, 2015; Kaiser and Schütz-Bosbach, 2018). In a series of studies, Daliri and Max observed a failure of AWS to attenuate auditory input (i.e., auditory suppression) before speech or listening to a sound, as indexed by N1 amplitude (Max and Daliri, 2019; Daliri and Max, 2016; Daliri and Max, 2015). A similar lack of sensory attenuation in AWS was also previously observed in the somatosensory domain (McClean, 1996).

Previous studies have similarly suggested a link between stuttering and a global inhibition of action (Neef et al., 2018; Arenas, 2017). However, unlike the view that inhibition is a learned response *to* stuttering (Orpella et al., 2024), the ideomotor perspective proposed here assumes inhibition to *be* the necessary and sufficient condition of stuttering. Although stuttering largely affects the domain of speech, the inhibitory phenomena underlying stuttering may hinder any form of communication facilitated by sequential perception-action cycles, including sign language (Cosyns et al., 2009; Whitebread, 2014) and handwriting (Fagan, 1932; Prasad and Pal, 2018). Performance pressure has been long known to disrupt the initiation of movement, with aberrant attentional mechanisms usually implicated as the culprit (Beilock and Gray, 2007; Abernethy et al., 2007). This form of ‘paralysis by analysis’ may occur when the efficient initiation of perception-action cycling underlying movement is inhibited, likely by excessive sensory precision (Harris et al., 2023; Cappuccio et al., 2020). Given communication often occurs under considerable social pressure, the development of communicative ‘paralysis by analysis’ is not surprising in at least a small proportion of speakers. This view of stuttering as inhibition has been long proposed (Bluemel, 1935) and is consistent with previous ideas of stuttering behavior stemming from the gradient activation of a behavioral inhibition system relative to the defensive distance and intensity of a perceived threat (Usler, 2022b).

The inhibitory mechanisms underlying stuttering may be alleviated or exacerbated depending on the precision dynamics between prior expectations and sensory input. Reducing sensory precision through the direct manipulation of sensory feedback should have an immediate and profound effect in reducing stuttering behavior (Saltuklaroglu et al., 2002). For example, auditory masking may also reduce sensory precision simply by the removal of the auditory input (Maraist and Hutton, 1957). A more amelioratory approach than reducing sensory input could be to make it one’s speech more predictable, such as through choral or shadowed speech (Charles Healey and Howe, 1987; Kalinowski and Saltuklaroglu, 2003). Similarly, delayed auditory feedback (DAF) and frequency-altered feedback (FAF) provide a simulated ‘second speech signal’ once the speaker self-initiates the initial syllable (Saltuklaroglu et al., 2004). Interestingly, these fluency-inducing effects are not limited to the auditory domain but can be solely visual (Kalinowski et al., 2000). Exogenous sensory stimuli, such as a ‘second speech signal,’ provide interpersonal entrainment to a speaker’s production, including timing cues for rhythmic synchrony, resulting in a highly predictable sensory environment. Beyond aiding exteroceptive predictions, this second signal may help stabilize interoceptive predictions (e.g., heart rate, respiration). Furthermore, ‘keeping together in time’ with others may affirm higher-level predictions regarding one’s social status within a larger group, resulting in positive affect (McNeill, 2009). Through these manipulations, increasing the predictability of one’s own sensory input may heighten prior precision while reducing attention (i.e., sensory precision) to one’s speech.

It has previously been proposed that the stuttering-reducing effects of choral speech, shadowed speech, DAF, and FAF may be due to the engagement of mirror neurons facilitating fluent imitation (Kalinowski and Saltuklaroglu, 2003; Saltuklaroglu et al., 2004). In support of this view, active inference provides a compelling framework for explaining the function of mirror neurons (Kilner et al., 2007a; Kilner et al., 2007b). Mirror neurons activate both when observing and performing the same action. It has been proposed that mirror neurons function within the generative model hierarchy, connecting higher-level predictions (e.g., goals) to lower-level sensorimotor predictions (Kilner et al., 2007a). Observing someone else’s action makes the sensory consequences of that action more predictable. When the environment is highly predictable, the generative model increases prior precision, and thus reduces precision afforded to the sensory input. This reduced prediction error leads to a decrease in the salience of environmental stimuli, resulting in less attention directed toward those stimuli. The more external stimuli reflect the expected sensory input, the greater the ameliorating effects on stuttering are likely to be (Kalinowski and Saltuklaroglu, 2003). As Kalinowski and Saltuklaroglu state: “*the closer the external gestural representation is to the intended utterance, the more likely it will be to enhance fluency*” (Kalinowski and Saltuklaroglu, 2003). How FAF, DAF, and masking directly manipulate sensory precision remains empirical questions for future investigation.

The interplay between active inference and the predictive capabilities of mirror neurons offers a valuable perspective on stuttering behavior and its modulation through external feedback. However, a deeper understanding of how these processes are underpinned by neural dynamics requires exploring the mechanisms of neural oscillations within the active inference framework. These oscillations facilitate the hierarchical integration of prior expectations and sensory input, offering insight into the precision-weighting mechanisms that influence stuttering behavior. Neural process theories of active inference involve neural message passing that correspond to the layers of the cortex and associated sub-cortical regions. Low-frequency neural oscillations, spanning delta to gamma bands, represent the summation of synchronous post-synaptic potentials and are thought to transmit prior expectations downward and prediction errors upward across the model hierarchy (van Kerkoerle et al., 2014; Bastos et al., 2012; Engel et al., 2001). These oscillations may represent distinct processes for predicting the timing of upcoming sensory events (Arnal et al., 2014), such as syllables and phonemes (Morillon et al., 2012; Giraud and Arnal, 2018; Arnal et al., 2011; Arnal and Giraud, 2012). Neural oscillations may provide a temporal scaffold for the integration of top-down prior expectations and ascending prediction error, with different oscillatory bands mediating predictive processing at different levels of the model hierarchy (Bastos et al., 2012; Friston et al., 2015; Adams et al., 2015). Top-down and bottom-up neural message passing map to slower (e.g., alpha or beta) and faster (e.g., gamma) brain rhythms, respectively (Arnal et al., 2014; Bastos et al., 2020; Alamia and Van Rullen, 2019).

Neural oscillations in the alpha (~8–12 Hz) and beta (~13–30 Hz) frequencies may play functionally distinct roles in prediction and regulating the precision of prediction errors (Bastos et al., 2012; Galeano-Otálvaro et al., 2024; Palmer et al., 2019). Alpha oscillations are thought to regulate precision weighting of prediction errors, mediating sensory precision by optimizing attention toward salient sensory stimuli (Bastos et al., 2012; Arnal and Giraud, 2012). Beta oscillations, on the other hand, may be associated with top-down motor predictions (Galeano-Otálvaro et al., 2024), movement initiation (Alegre et al., 2004), sensory gating (Arnal, 2012), and internal timing (Cirelli et al., 2014). Typically, beta power decreases (i.e., beta suppression) before movement and rebounds after movement cessation (Kilavik et al., 2013; Neuper et al., 2006). Stronger beta suppression is linked to faster, well-learned, automatic movements (Tzagarakis et al., 2019), whereas reduced suppression may indicate imprecise sensory predictions (Kilavik et al., 2013). Beta power may index motor prediction uncertainty (Neuper et al., 2006), with suppression decreasing under uncertain conditions (Tzagarakis et al., 2010; van Helvert et al., 2021). Additionally, beta suppression at movement initiation has been associated with sensory attenuation (Engel and Fries, 2010) and post-movement beta rebound may reflect prediction error (Tan et al., 2014; Tan et al., 2016; Torrecillos et al., 2015; Torrecillos et al., 2018) and their associated uncertainty (Tan et al., 2016). Similarly, Palmer et al. demonstrated that pre-movement beta suppression is influenced by precision-weighting of prediction errors (Palmer et al., 2019), further supporting the link between beta suppression and uncertainty in movement preparation (van Helvert et al., 2021; Tzagarakis et al., 2021). From an active inference perspective, beta suppression may occur when the brain updates prior expectations in response to prediction error. Thus, a reduction in beta power at the initiation of action may index an increase of prior precision and decrease in sensory precision.

Fluent speech relies on timely beta suppression for smooth syllabic transitions. However, studies on neural oscillations in people who stutter (PWS) indicate atypical alpha (~8–12 Hz) and beta power during speech preparation and execution (Orpella et al., 2024; Jenson et al., 2019; Korzeczek et al., 2022; Mersov et al., 2016; Mock et al., 2016; Mollaei et al., 2021). Korzecek et al. found that stuttering severity correlated with increased alpha power in frontal regions and beta power in central areas during speech intention (Korzeczek et al., 2022). Similarly, Orpella et al. observed elevated beta power in the right pre-supplementary motor area before stuttered utterances, with severity-dependent variations (Orpella et al., 2024). Jenson et al. reported weaker alpha and beta suppression in the left premotor cortex during single word production (Jenson et al., 2018). Mersov and colleagues, however, found stronger beta suppression in the bilateral mouth motor cortex during both speech planning and execution of phrases (Mersov et al., 2016). According to Brown and colleagues, this discrepancy may be task-dependent (Brown et al., 2025): compared to non-stuttering peers, PWS may exhibit less beta suppression during simpler or more automatic speech (which relies less on feedback) but more alpha and beta suppression in more complex speech tasks that induce greater uncertainty (Jenson et al., 2018; Brown et al., 2025; Caruso et al., 2023). The proposed roles of alpha and beta oscillations in active inference, and the fact that their oscillatory dynamics approximate to speech initiation appear to be atypical in PWS and allow one to speculate about their role of precision dynamics in stuttering behavior.

## 6 Two hypotheses for a proximate cause of stuttering behavior

**Figure 3:**
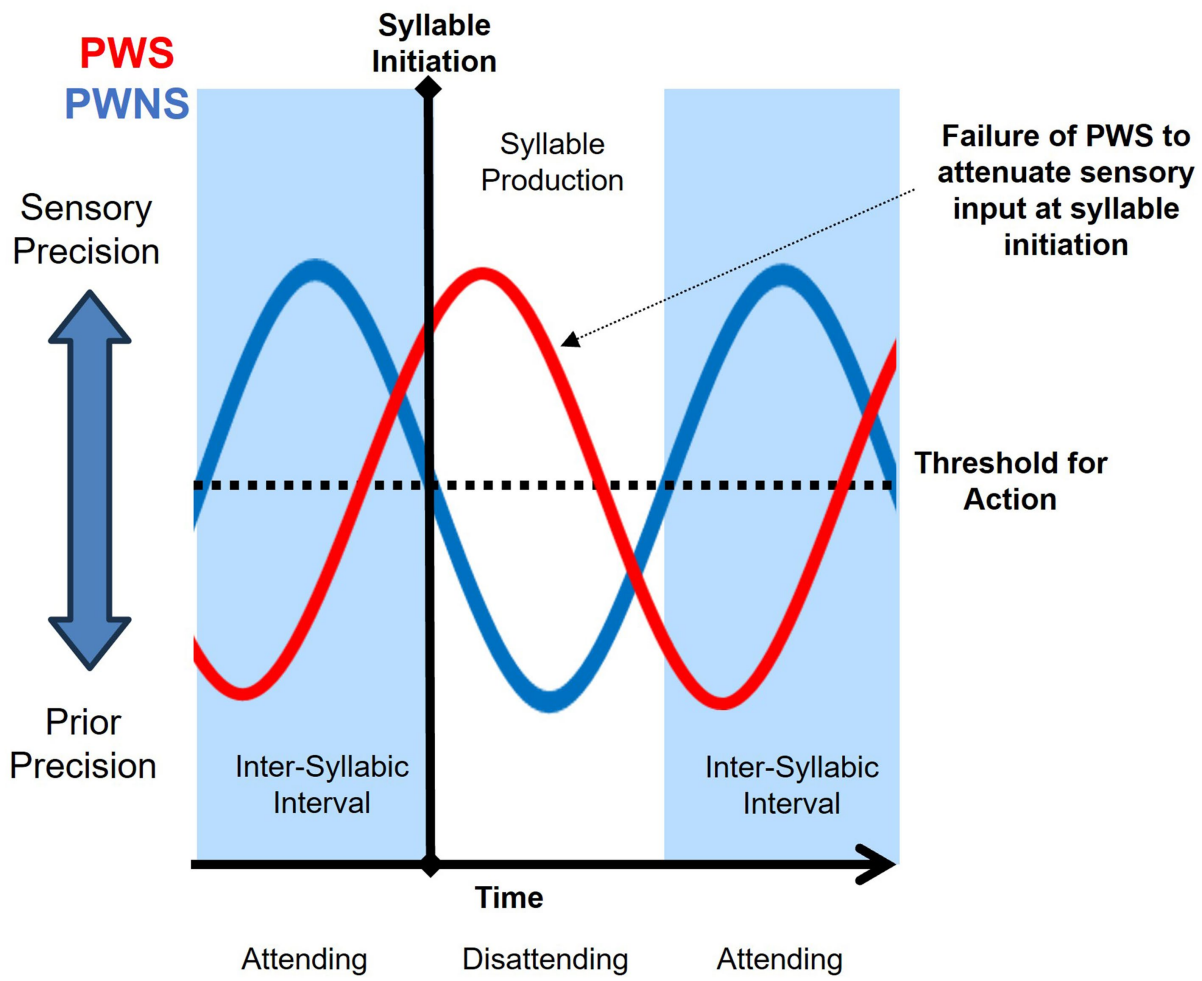
Mistiming hypothesis purports a phase shift in precision dynamics as a failure to attenuate sensory precision at syllable initiation of people who stutter (PWS; red) and typical syllable initiation by people who do not stutter (PWNS; blue).

Linking precision dynamics, sensory attenuation, and neural oscillations present a complicated and unfinished explanation of stuttering behavior, but circumstantial evidence supports two potential mechanisms for a failure of sensory attenuation at the onset of speech: (1) a phase shift in the precision dynamics relative to the timing of speech initiation (‘mistiming hypothesis’); or (2) excessive inward attentional focus at speech initiation (‘attention hypothesis’). The *mistiming hypothesis* implicates aberrant temporal scheduling of the modulation of sensory and prior precision, resulting in a lack of sensory attenuation at syllable initiation (See Figure 3). When syllable initiation is attempted, sensory precision has yet to decrease past a threshold for action. This scenario could be caused by a specific difficulty in predicting the timing of sensory inputs, which is similar to previous accounts of stuttering as a deficit in temporal processing (Sares et al., 2019; Falk et al., 2015). Stuttering has been associated with disruptions in sensorimotor integration, speech timing, and speech motor performance (Smith, 1999; Alm, 2004; Etchell et al., 2014). The speech of PWS is also more fragmented and less rhythmic than that of their non-stuttering peers (Yawatkar et al., 2023; Boecher et al., 2025). Decreased functional activity and structural connectivity in neural networks that support the timing of self-paced movement, such as between the left putamen, left inferior frontal gyrus, and the middle temporal gyrus, have been associated with stuttering (Chang and Zhu, 2013). In an investigation of neural activity in the left precentral gyrus, Mersov and colleagues observed non-stuttered utterances to be facilitated by an earlier (~200 ms) onset of beta suppression compared to stuttered utterances (Mersov et al., 2018). In a study by Etchell and colleagues, aberrant entrainment of beta power was observed in CWS during passive listening to tones (Etchell et al., 2016). Typically developing children exhibited peaks in beta power approximate to the sound onset. However, beta peaked ~200 ms after sound onset for CWS. Etchell et al. (2016) concluded that, unlike their typically developing peers, beta modulation of CWS appeared “reactive rather than predictive”. Although greater empirical research is needed, the findings of Mersov et al. (2018) and Etchell et al. (2016) implicate a potential mistiming in precision dynamics associated with stuttering behavior.

If this *mistiming hypothesis* is true, any circumstance that fosters synchrony between sensory attenuation and syllable initiation would reduce stuttering behavior. Speaking to the beat of an external rhythm (e.g., metronome or choral speech) tends to reduce the likelihood of stuttering [For review, see Bloodstein et al. (2021)]. Providing rhythmic cues for the speaker to initiate speech may aid temporal prediction of sensory input, resulting in sensory attenuation coordinated with syllable initiation. Relatedly, rhythmically moving parts of the body during speech may serve as a self-generated cue (Barber, 1940). Imposing a slight delay (i.e., pause) to syllable initiation may also afford time for sensory attenuation to cross the threshold for action and allow for fluent production. Inserting short pauses before speech initiation has been considered a helpful therapeutic technique for many PWS (Reitzes, 2006). Alternatively, imposing a delay to incoming sensory input within the perception-action cycle (i.e., ~50–200 milliseconds) may minimize the aberrant phase shift in precision dynamics, and could facilitate sensory attenuation under the threshold necessary for syllable initiation. This phenomenon has been long observed with the use of DAF in the amelioration of stuttering [For review, see Bloodstein et al. (2021)]. Interestingly, a lack of auditory suppression previously observed in AWS was normalized by a 100 ms delay in auditory feedback (Max and Daliri, 2019). On the contrary, delay in auditory feedback may prevent timely sensory attenuation in those who typically do not stutter, resulting in ‘stutter-like’ behavior (Corey and Cuddapah, 2008).

**Figure 4:**
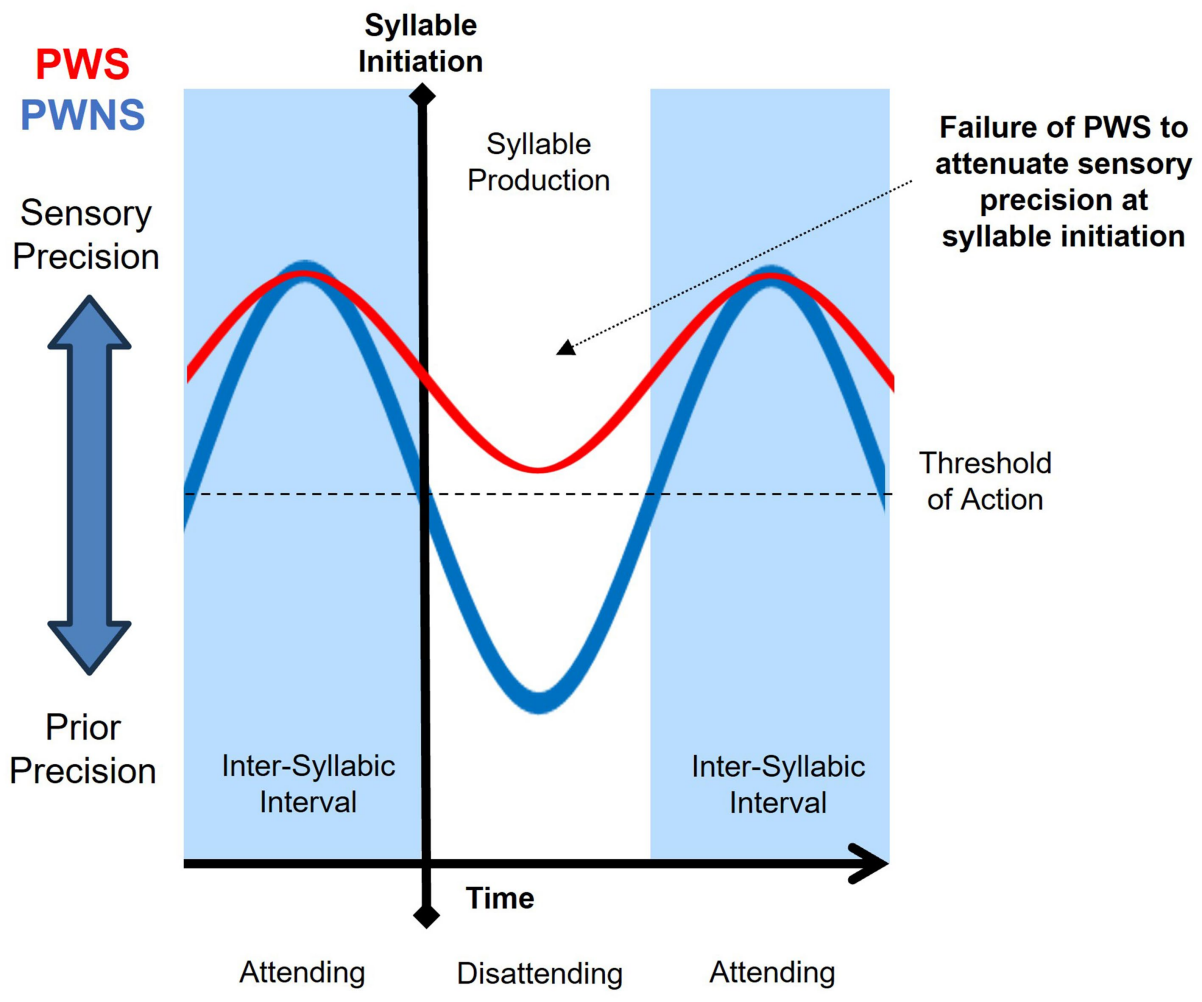
Attention hypothesis purports increased attention (or lack of disattending) sustains relatively high sensory precision, resulting in a failure of sensory attenuation by people who stutter (PWS; red) relative to people who do not stutter (PWNS; blue).

The *attention hypothesis* implicates a lack of sensory attenuation immediately prior to syllable initiation without atypical timing (See Figure 4). In this case, the magnitude of sensory precision is simply not attenuated past a threshold necessary to initiate action. This lack of sensory attenuation may be due to excessive selective attention to the act of speaking or inability to disattend to current sensory input during speech (Brown et al., 2013; Limanowski, 2017). Functional motor disorders have been associated with strong sensory precision due to excessive inward attention to the self (Pareés et al., 2014). Not surprisingly, stuttering behavior is a common sign of functional speech disorders (Utianski and Duffy, 2022; Baker et al., 2021). Increased attention is deployed to resolve uncertainty by affording greater salience to observations as a function of their information content. The types of utterances that are usually stuttered, described in Section 4, are those that resolve uncertainty (i.e., high information content) and thus made more salient by the speaker’s attention. Beyond the language spoken, circumstances that induce increased attention to the self as speaker increase the likelihood of stuttering (Maddox, 1938; Kamhi and McOsker, 1982).

If the *attention hypothesis* is true, disattending to current sensory input during speech should reduce stuttering, without any alteration in speech timing. Stuttering does not typically occur when communicative environments reduce sensory precision, as observed in the effects of distraction, altered auditory feedback, and contingent stimuli [For review, see Bloodstein et al. (2021)]. Many PWS rely on forms of self-distraction to prevent or alleviate stuttering behavior. In the case of FAF and auditory masking (e.g., white noise), sensory precision afforded to speech may be directed elsewhere or reduced altogether. Given the ideomotor nature of active inference, the effectiveness of suggestion on reducing stuttering behavior should not be surprising (Bloodstein et al., 2021; Bloodstein, 1949). Suggestion may reduce sensory precision to the act of speaking by altering attentional processes whether the suggestion is hypnotic or non-hypnotic, imposed or self-induced (Raz and Campbell, 2011). Similarly, any sense of novelty or deviation from how one traditionally speaks, such as performing a role or changing vocal characteristics, may be viewed as a form of disattending from the self as speaker (Bloodstein et al., 2021; Bloodstein, 1949). Dual-task paradigms have also revealed that stuttering may reduce if attention is orientated towards other sensory stimuli (Vasic and Wijnen, 2005; Arends et al., 1988), and this effect may be particularly strong if attention is oriented towards sensory input from rhythmic limb movement synchronized to speech (Barber, 1940).

Although these two hypotheses suppose different causal mechanisms, they are likely not mutually exclusive and may even reinforce each other. For example, PWS exhibit considerable individual differences in responding to altered auditory feedback (e.g., DAF/FAF) and, on average, these interventions all appear to be effective at ameliorating stuttering behavior (Lincoln et al., 2006). The novelty of any alteration of natural speech as a potential form of self-distraction supports the *attention hypothesis*. Yet the *attention hypothesis* does not explain how DAF elicits stutter-like behavior in people who typically do not stutter (Corey and Cuddapah, 2008). Further evidence for either hypothesis includes previous associations between stuttering and deficits in the processing of temporal information, including errors in time estimation (Bastos et al., 2012). PWS may overestimate in their perception of time and stuttering behavior has been positively correlated with time estimation difficulty (Ezrati-Vinacour and Levin, 2001; Barasch et al., 2000). Previous research supports the notion of increased attention as prolonging the perception of time, which would support the *attention hypothesis* [For review, see Tse (2010)]. However, this subjective time dilation has also been found to increase with temporal uncertainty (Zhang et al., 2023). This finding and observed deficits in rhythm discrimination (Chang et al., 2016) and production (Boecher et al., 2025) in PWS gives credence to the *mistiming hypothesis*.

In sum, both hypotheses help to explain how the precision of current sensory input may fail to attenuate relative to that of prior expectation, resulting in the inhibition of syllable initiation. It is possible the stuttering behavior of some PWS, such as those who respond most favorably to DAF, are driven by mistiming of perception-action cycles. The stuttering behavior of other PWS, such as those who respond more favorably to FAF or masking, may be driven by excessive attention to their speech. Given precision dynamics are likely influenced by the degree of uncertainty associated with personal history, sense of self-efficacy, temperamental differences, and daily stresses, it is to be expected that the appearance of stuttering vary naturally between and within individuals who stutter, while also being somewhat ecologically predictable (Sheehan, 1969).

## 7 Loss of agentic control over speech

Overt stuttering spans a wide range of aberrant and involuntary behaviors. Most prominent are speech disruptions in the form of blocks (e.g., involuntary halts or fixed articulatory postures), prolongations (e.g., lengthening sounds), and repetitions (e.g., repeating sounds, syllables, or words) that develop in early childhood (Bloodstein et al., 2021). Stuttering has also been associated with increased tension and struggle during speech production, resulting in a felt experience of effortfulness and a ‘loss of control’ in speech (Perkins, 1990; Tichenor and Yaruss, 2019). During moments of stuttering, many PWS also exhibit aberrant low-frequency tremor in speech articulators and dystonic movements, such as facial grimaces, eye blinks, and head jerks (Kelly et al., 1995; Kiziltan and Akalin, 1996). As described above, precision estimation reflects the weight given to prior expectations or sensory stimuli. Precision also operates across multiple hierarchical levels for the initiation of action—shaping goal setting and policy selection at higher levels and movement segmentation and kinematics at lower levels (Limanowski et al., 2024; Proietti et al., 2023). Experimental manipulation of precision dynamics at different levels of this hierarchy is currently lacking, but computational simulations by Parr and colleagues are suggestive that a failure to attenuate sensory precision at the level of policies induces akinesia or freezing of motor initiation, akin to Parkinson’s disease (Parr et al., 2021). This study also distinguishes spatial and temporal precisions—spatial precision refers to the confidence in estimating the position of effectors in space. In contrast, temporal precision is the confidence afforded to the timing of sensory input. In simulating limb movement, Parr et al. found that not attenuating spatial precision disrupted motor reflexes akin to motor neuron pathologies, while imprecise temporal precision led to disrupted movement coordination. Future work is necessary to determine if these computational findings are relevant in explaining disordered speech motor control. One may speculate that specific disruptions in neuromuscular activation for speech may be associated with aberrant spatial precision (e.g., spastic dysarthria) and temporal precision (e.g., ataxic dysarthria). Aberrant precision dynamics in the continuous inference underlying movement trajectories may explain many speech-motor pathologies; however, this low-level neuromuscular function is largely unimpaired in those who stutter, particularly children soon after onset (Walsh and Smith, 2013; Smith and Luschei, 1983). Instead, the relatively late development, situational variability, and linguistic loci of stuttering behavior implicate stuttering as a disorder of aberrant policy precision—a difficulty in planning syllable sequences to optimally minimize expected precision error across longer movement sequences. Such a disorder would implicate inefficiencies in the basal ganglia and associated cortical areas in estimating and selecting optimal policies through dopaminergically-modulated policy precision (Friston et al., 2012). Over time, chronic disruption to the discrete inferential processes of the basal ganglia may impair neural message passing to lower-level inferential processes driving continuous muscular activation and kinematics (Parr et al., 2021). For example, a subset of young CWS develop aberrant neuromuscular pathologies, such as tremor of the lips and jaw, in later childhood and adolescence (Kelly et al., 1995). The disruptions in policy selection and syllable sequencing that underlie stuttering behavior often give rise to broader behavioral adaptations aimed at mitigating or avoiding stuttering over longer timescales.

Stuttering behavior over longer timescales may be viewed as cognitive and behavioral reactions to an attempt to prevent or avoid the more transient behaviors mentioned above, such as circumlocution, word substitution, and avoidance [For review, see Bloodstein et al. (2021)]. For instance, consider a speaker entering a communicative situation and knowing they will have to say a word that they often stutter on. They are likely to be uncertain in their ability to fluently traverse the linguistic landscape (i.e., *Will I stutter or not? What course of action should I take to make stuttering less likely?*). These cognitions and behaviors that extend beyond the perception-action cycle may be the product of, and contribute to, atypical precision dynamics afforded to syllable transitions and policies that unfold over longer timescales, from phrases to entire conversations. Current active inference models (e.g., Markov decision process) include precisions associated with policies (i.e., policy precision) that transition an agent from one state to the next (i.e., transition precision) (Fradkin et al., 2020). This lack of confidence in transitioning from one syllable to the next is felt as a series of momentary uncertainties analogous to walking a tightrope. In other words, greater uncertainty when transitioning from one syllable to the next or when choosing the appropriate syllable sequence is likely to increase attention to the current sensory input. This dynamic has been previously described as anticipatory struggle (Bloodstein, 1972). On the contrary, the less sensory precision interferes with syllable initiation, the greater transition precision will be afforded when moving from one syllable to the next. Stuttering behavior largely occurs on the initial syllable of an utterance because higher uncertainty fosters deployment of selective attention towards high information content, thus boosting sensory precision (Taylor, 1966; Goldman-Eisler, 1958). As the speaker traverses the linguistic landscape, particularly during open discourse, speech exhibits a decreasing information rate (Giulianelli and Fernández, 2021), resulting in less likelihood of stuttering across an utterance (Quarrington, 1965). This is consistent with Sheehan’s fear reduction hypothesis (Sheehan, 1953), may be due to the previous stuttering behavior removing the speaker’s attention away from the next syllable.

If stuttering becomes chronic, communicative apprehension may grow such that a speaker learns to mistrust their overall ability to minimize expected prediction errors into the future. For instance, the speaker becomes uncertain about how to transmit the larger message, resulting in word substitutions and circumlocutions, that indicate a loss of agentic control over the communicative environment (Perkins, 1990). This uncertainty in selecting the best policy, or sequence of actions, is reflected by low policy precision (Sandved-Smith et al., 2021). Chronic disruption to perception-action cycling may induce a vicious cycle of attentional capture to specific sounds, words, and situations that further increases sensory precision that is causal to stuttering behavior (Kessler, 2016). When stuttering occurs on a particular word, greater attention to that word will increase sensory precision in future scenarios and this may be felt as premonition of future stuttering (Jackson et al., 2019; Cholin et al., 2016). This phenomena fuels the long-known ‘consistency’ and ‘adjacency’ effects in which stuttering predictably occurs across repeated utterances (Tate and Cullinan, 1962; Avari and Bloodstein, 1974). This chronic and transient increase in sensory precision via attention is analogous to previous theories linking stuttering to excessive self-monitoring during speech production (Bernstein Ratner and Wijnen, 2007). The environmental unpredictability that comes with the variability of stuttering may lead to persistent uncertainty and prediction errors. If the agent cannot act to reliably reduce prediction error, either because of weak prior expectations or because the environment is too volatile, a lack of control or agency may emerge. If the model consistently assigns excessive precision to incoming sensory signals, a state of heightened arousal or vigilance may become habitual. Agentic loss of control over communicative ability can be viewed in active inference as reduced transition and policy precision to such a degree that acting for information gain (i.e., exploration) is deemed too risky. Instead, expected prediction error is minimized by limiting goal-directed action. For PWS, the perceived perils of stuttering can lead one to avoid speaking and/or choose a lifestyle centered on avoiding potential stuttering (i.e., covert stuttering). This strategy is effective at minimizing uncertainty in the short-term, but at the expense of long-term wellbeing because information gain is necessary for model updating in a dynamically changing world. This paradox of choosing between approach and avoidance is analogous to the ‘dark room problem’ (Van de Cruys et al., 2020; Friston et al., 2012). Inversely, uncertainty reduction during speech is likely to be fostered through active exploration of the world.

One can speculate that this shift in relative precision from sensory input to prior expectation facilitates the ‘adaptation effect’ in which PWS exhibit a gradual reduction in disfluencies when repeatedly reading or speaking the same passage within a short timeframe (Harris, 1942; Wingate, 1986). With repeated speech, prior expectations are strengthened, and uncertainty is reduced. This may lower attention-moderated sensory precision and increase policy precision as speech production becomes more automatic. Thus, the adaptation effect emerges as speech transitions from a high-uncertainty, effortful process to a predictable, automatic one. More generally speaking, this increased confidence (in prior expectation) has been expressed by PWS who actively communicate regardless of occasional stuttering behavior (Finn and Felsenfeld, 2004). This idea of openly stuttering and ‘saying what one wants to say’ has been clinically operationalized by therapeutic programs for stuttering (Byrd et al., 2021; Sisskin, 2018). Conversely, feelings of stress or anxiety may increase the likelihood of stuttering by increasing an inward and negatively-valanced self-consciousness during speech production, which is likely to boost sensory precision precisely on those words that are most feared or anticipated for stuttering to occur. Stress increases epistemic uncertainty during speech planning, making speech less automatic and more effortful, reinforcing the cycle of self-monitoring and increased disfluency.

Chronic stuttering behavior also negatively impacts well-being beyond speech and language (Jacobs et al., 2021; Pruett et al., 2021). Metabolic processes regulate energy production and expenditure to keep the brain functioning within an optimal range, particularly during periods of stress (Peters et al., 2017). By minimizing uncertainty, active inference optimizes energy utilization and reduces metabolic costs (Barrett et al., 2016). The brain is largely efficient in its energy expenditure during periods of low uncertainty via optimal precision weighting facilitated by synaptic transmission (Harris et al., 2012). Conversely, the processing demands that come with selective attention and boosting sensory precision during uncertainty are metabolically expensive (Peters et al., 2017). This energy expenditure is felt as cognitive effort—the cost of frequently updating prior expectations in response to frequent prediction error (Parr et al., 2023; Zénon et al., 2019). For many people, speaking involves minimal cognitive effort because they rely on precise policies (i.e., you know what you want to say) based on precise transitions (i.e., you easily move from one syllable to the next) and precise prior expectations (i.e., you hear what you expected). For PWS, speech is computationally and metabolically expensive. It was recently found that CWS exhibit greater gray matter volume, compared to non-stuttering children, in brain areas correlated with gene expression involved with metabolic function. This suggests a relationship between energy metabolism and stuttering in children (Boley et al., 2021; Chow et al., 2020). Furthermore, many PWS exhibit gene mutations associated with lysosomal enzyme trafficking (Han et al., 2019). Although it remains unknown how these mutations and lysosomal storage abnormalities relate to stuttering, it has been proposed that a deficiency in lysosomal function may result in reduced brain metabolism or neural energy supply (Alm, 2021a; Sheikh Bahaei et al., 2023). Chronic interoceptive experience of disfluency is felt as fatigue, anxiety, and perhaps even depression (Stephan et al., 2016). For many PWS, quality of life is reduced by diminished vitality, increased fatigue, and other depressive symptoms (Bills, 1934; Craig et al., 2009). This cost is likely to be particularly acute for PWS who rely on continual covert actions and avoidance behavior to ‘pass as fluent’ (Constantino et al., 2017). Speech production can be both cognitively and metabolically taxing for PWS, with inefficiencies in prediction error minimization leading to effortful speech and decreased overall quality of life. This underscores the importance of fluency in everyday functioning, particularly speech production.

Fluency can be understood as the process in which model accuracy and complexity are balanced to achieve optimal prediction error minimization via perception-action cycling. As a result, fluent movements are perceived as smooth, graceful, and effortless, at the price of minimal computational and metabolic cost. This concept aligns with theories in cognitive science, linguistics, and psychology, where fluency reflects mastery and efficiency of cognition and action. The framework of active inference, a corollary of the free energy principle, is conceptually similar to the minimization of the Lagrangian (i.e., difference between kinetic and potential energies) over time in classical mechanics. This fundamental aspect of nature, the *principle of least action*, ensures that the dynamics of physical systems are such that the ‘action’ is minimized or stationary [for a review of this principle, see Gray (2018)]. For our purposes, fluency can be conceptualized as the enactment of the principle of least action for communicative behavior, akin to Zipf’s principle of least effort (Zipf, 2016). Stuttering, as a disorder of fluency, may reflect a chronic inability to minimize prediction error, resulting in disrupted transitional flow in perception-action cycling. As a result, stuttering manifests as the involuntary, transient, and habitual inhibition of syllable initiation that makes speech effortful, fragmented, often accompanied by physical tension or compensatory behaviors, which may paradoxically exacerbate the symptomology akin to Huxley’s law of reversed effort (Huxley, 2021). These issues may stem from deeper neural mechanisms, implicating neuromodulatory systems.

## 8 Stuttering as synaptopathy

**Figure 5:**
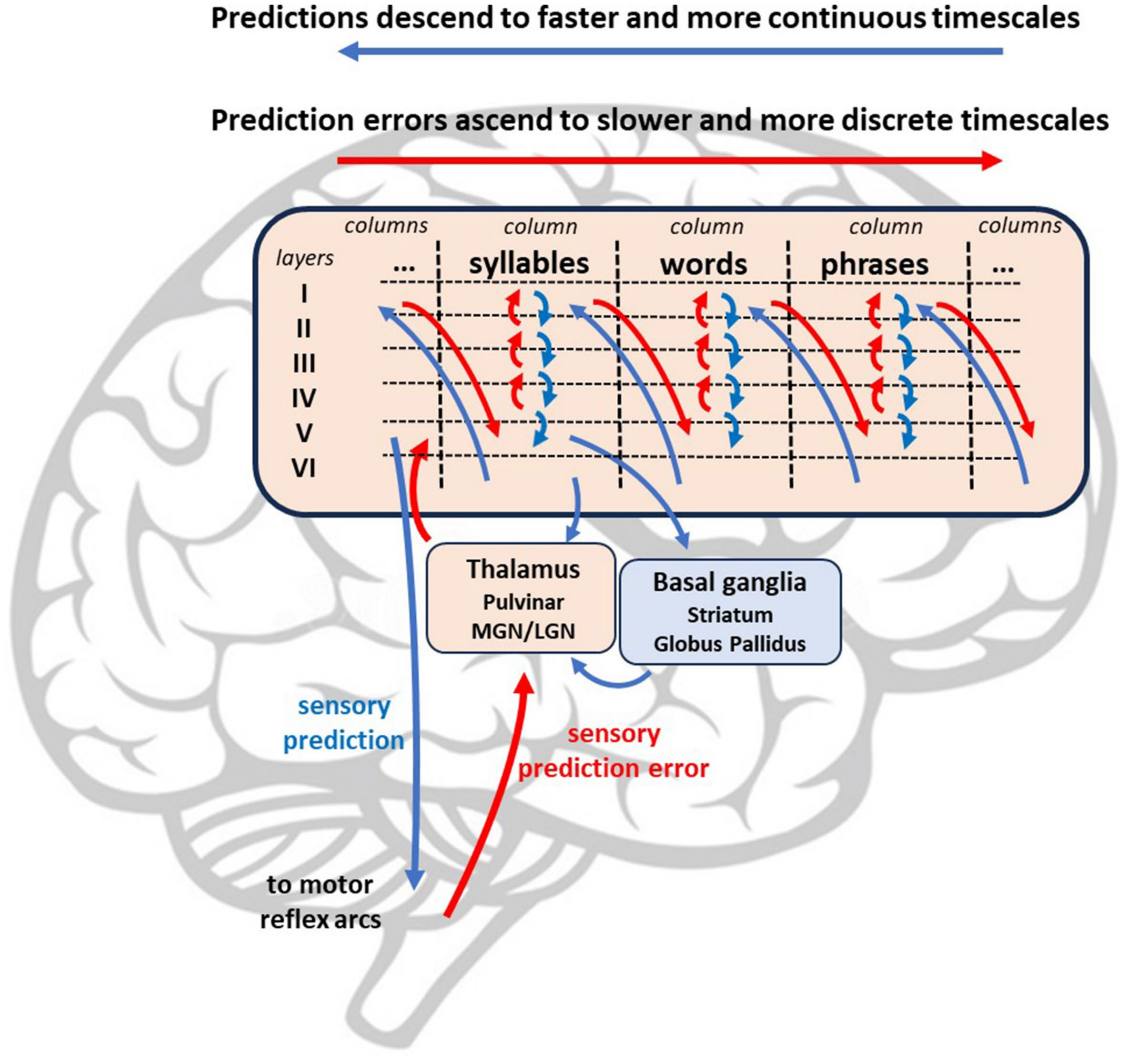
Simple hierarchical model with lower levels including subcortical areas processing ascending sensory prediction error (red) which are resolved by multiple levels of descending predictions (i.e., prior expectations).

As stated above, the active inference account of stuttering proposed above maps onto hierarchical and temporally deep message-passing models of the brain (Parr et al., 2021; Parr and Friston, 2018). Sensory prediction errors ascend hierarchical subcortical pathways from the inferior colliculus to the thalamic nuclei (e.g., ventral posterior nucleus for proprioceptive prediction error and medial geniculate nucleus for auditory prediction error) (Carbajal and Malmierca, 2018). Upon reaching layer IV of the cortex, prediction errors are resolved by prior expectations at higher cortical levels (I-III), with this inferential processing becoming more categorical and abstract as residual prediction errors move anteriorly to the prefrontal cortex (Carbajal and Malmierca, 2018; Escera, 2023). Neural circuits at higher (i.e., more anterior) levels of the model encode sequences of information from lower levels, such as syllables, words, and phrases (See Figure 5). Predictions are sent via upper motor neurons from cortical layer V to the brainstem and spinal cord. Within closed-loop motor reflex arcs, this prediction is subtracted from the incoming proprioceptive input, resulting in prediction error that is minimized by movement.

This neural network is supplemented by connections between the cortex, thalamus, and basal ganglia that assist with the computation of precision-weighting, expected prediction error, and the selection of optimal policies. A connection between the cortex and the thalamic nuclei (i.e., pulvinar) mediates the precision-weighting of ascending prediction errors (Kanai et al., 1668). Predictions are also sent from layer V to the basal ganglia, which is divided into two pathways: the direct pathway, which activates (i.e., disinhibits) thalamic signaling to the motor cortices, and the indirect pathway, which inhibits activation. The direct pathway, which includes the striatum and internal globus pallidus, may compute expected prediction error of a particular policy. The direct pathway promotes goal-directed action by selecting policies with relatively low expected prediction error. The indirect pathway counters this motivation by suppressing implausible policies and promoting more conservative policies (i.e., habits) based on previous experience. These two pathways combine at the internal globus pallidus and send select optimal policies to the thalamus (Parr, 2020).

Current theories of predictive processing have implicated neuromodulation as the key mechanism underlying precision dynamics (Parr et al., 2021). Acetylcholine, norepinephrine, and dopamine have all been associated with precision parameters and have all been associated with stuttering behavior (Ekhart et al., 2021). Norepinephrine, a neurotransmitter that modulates transition precision during unexpected uncertainty (Parr and Friston, 2018; Zhao et al., 2018), has been shown to increase significantly in PWS during speech, perhaps due to increased sympathetic activation (Edgren et al., 1969). Dopamine is a neurotransmitter that encodes reward prediction error (Schultz et al., 1997). From an active inference perspective, dopamine modulates policy precision and sets the balance between the direct and indirect pathways in inferring which policies should and should not be selected, respectively (Parr, 2020). Stuttering has been primarily associated with excessive dopaminergic signaling in the basal ganglia (Maguire et al., 2002; Maguire et al., 2020; Wu et al., 1997). However, stuttering may more generally occur if dopamine deviates from an optimal range (Sheikh Bahaei et al., 2023; Ekhart et al., 2021; Alm, 2021b). Overly precise policies due to a hyperdopaminergic state can result in overly fixed movement patterns, including cognitive inflexibility and perseverative behavior (Berridge et al., 2005). Excessive dopamine may reflect high precision in a chosen policy, which reduces prediction error in subcortical auditory pathways, including the inferior colliculus (Valdés-Baizabal et al., 2020). Conversely, a hypodopaminergic state and under-precise policies can lead to effortful and slowed movement (Bologna et al., 2020).

Acetylcholine has been implicated as a neuromodulator of sensory precision (Pérez-González et al., 2024). The prefrontal cortex connects to the basal forebrain, which releases acetylcholine to modulate sensory gain in response to prediction error (Yu and Dayan, 2002). A reciprocal interaction between dopamine and acetylcholine within the basal ganglia for the control of movement has been long known, with dopamine regulating the release of acetylcholine in the striatum (Surmeier and Graybiel, 2012). Activation of striatal cholinergic interneurons can also elicit dopamine release (Shin et al., 2015). Although their dual influence is complex and likely context-specific, an imbalance between dopamine and acetylcholine may underlie at least some aspects of stuttering behavior (Ekhart et al., 2021). It is clear that much needs to be done to understand how neuromodulatory systems and their interactions may influence stuttering behavior. Although neural process theories based on active inference principles are currently in infant stages and highly speculative, stuttering may be viewed as a synaptopathy resulting from atypical precision dynamics (Friston, 2023).

## 9 Current limitations, future directions, and clinical implications

Applying principles of active inference to stuttering does reveal several limitations. One major issue is the complexity of stuttering etiology. Recent work on the etiology of stuttering has identified potential neurobiological abnormalities across numerous levels of analysis [For review, see Sheikh Bahaei et al. (2023)]. Stuttering is highly influenced by genetic, biological, and even social factors, which are not fully addressed, or have yet to be addressed, by active inference models. Additionally, the neural mechanisms underlying stuttering, such as those in the basal ganglia and motor cortex, remain unclear in this framework. Little is understood about the effects of the corpus callosum and hemispheric lateralization, as well as the role of numerous subcortical areas, such as the amygdala and cerebellum. These brain regions are central to speech motor control and stuttering (Guenther, 2016), but have yet to be widely incorporated into active inference models. Other models, such as the DIVA model, have a more comprehensive set of neurophysiological correlates. Unlike the DIVA and HSFC models, a well-developed model of speech production that explains articulatory kinematics based on the principles of active inference has yet to be fully developed. This proposed account of stuttering behavior is limited by the nascent state of the concept of active inference and related neural process theories.

The functional role of neural oscillations within the active inference framework is also currently unclear, with evidence suggesting these low-level oscillations may play a role in transmitting prediction and prediction error across the generative model. Although there is considerable circumstantial evidence that atypical low-frequency oscillations associated with speech preparation and production are associated with stuttering behavior, more research is necessary to better understand this relationship. Determining the neural correlates of precision dynamics is difficult because it likely involves the integration of different modalities (e.g., exteroception, interoception, proprioception), as well as combining the precisions of prior expectations and sensory input. It is important that the theoretical assumptions of active inference are not contingent on any particular theory of neural mechanisms.

Active inference provides future opportunities for empirical validation through discrete and continuous computational modeling, largely using partially observable Markov decision process (POMDP) models (Smith et al., 2022). POMDPs evaluate action sequences based on their likelihood of achieving desired outcomes. At the same time, continuous models translate these plans into action, bridging higher-level discrete cognitive and linguistic processes with lower-level motor processes that drive the movements of muscle and bone. Recent computational work, largely in the domains of active vision (Parr et al., 2019) and active listening (Friston et al., 2021) may be translated into computational modeling of (disordered) speech production. These models, using discrete time steps and variables, provide a formal framework for testing active inference assumptions.

Future modeling of speech perception and production based on active inference will likely increase our understanding of the relationship between precision dynamics and stuttering behavior in ways that inform the clinical interventions of stuttering that alter precision dynamics across various levels of the hierarchical model. Any protocol that enhances the predictability of the environment, thereby reducing uncertainty and sensory precision, via cognitive behavioral training or feedback modulation, could recalibrate aberrant precision dynamics to reduce the elicitation of stuttering behaviors. Behavioral approaches that aim to re-structure speech, such as ‘fluency shaping’ or ‘stuttering modification’ (Guitar and McCauley, 2009), and more recent approaches that focus on effective communication, such as Avoidance Reduction Therapy for Stuttering (Sisskin, 2018) and CARE Model of Treatment for Stuttering (Byrd et al., 2024), could be reframed as strategies that foster precise prior expectations of communicative competence up the model hierarchy. More immediate techniques that directly alter current sensory feedback (e.g., masking, DAF/FAF, etc.) can also be effective at manipulating sensory precision at syllable initiation, that often dramatically alleviates stuttering behavior. However, these immediate positive effects are likely to dissipate without a concordant effort to increase prior precision of sensory consequences of speech. Alternately, the use of psychedelics may alleviate stuttering behavior in some individuals (Jackson et al., 2024) by decreasing high-level prior precisions regarding one’s communicative abilities in accordance with the Relaxed Beliefs Under Psychedelics (REBUS) model (Carhart-Harris and Friston, 2019). Simply put, stuttering may only be ameliorated in the long term by developing high-level prior expectations that stuttering is not likely to occur (Finn and Felsenfeld, 2004).

## 10 Conclusion

Despite extensive scientific investigation over the past century, the complex and functional nature of stuttering has thwarted attempts at a parsimonious explanation. Active inference is a predictive processing account of sentient behavior that may help to explain the etiology and phenomenology of stuttering behavior. Stuttering may arise from atypical sensory precision and prediction error dynamics, inhibiting syllable initiation. High sensory precision, resulting from mistimed precision dynamics or excessive attentional focus can impede the sensory attenuation required for fluent speech initiation. Reframing stuttering as a synaptopathy integrates neurobiological, psychological, and behavioral dimensions, suggesting disruptions in precision-weighting mediated by neuromodulatory systems like dopamine and acetylcholine. As a result, the development of aberrant perception-action cycling leads to a vicious cycle that disrupts speech fluency.

## Data Availability

The original contributions presented in the study are included in the article/supplementary material, further inquiries can be directed to the corresponding author/s.

## Glossary

Acetylcholine: A neurotransmitter involved in modulating sensory precision and attentional focus.
Active Inference: A theoretical framework in neuroscience and cognitive science describing agents minimize the difference (or free energy) between their generative modal and the environment by perception and action.
Adjacency Effect: The tendency for stuttering to occur near previously stuttered words in repeated speech trials.
Adaptation Effect: The phenomenon where repeated readings or speaking of the same text lead to a reduction in stuttering.
Allostasis: The process by which the brain and body maintain stability by predicting and adapting to environmental demands.
Attention: The cognitive process of selectively focusing on certain stimuli while ignoring others, which influences sensory precision and prediction error minimization.
Basal Ganglia: A group of subcortical brain structures involved in motor control, speech production, and decision-making, often linked to stuttering.
Bayesian Inference: A statistical method where prior knowledge (beliefs) is updated with new evidence to generate a posterior belief.
Bayesian Priors: Pre-existing beliefs or expectations that influence how new sensory information is interpreted.
Cholinergic System: The neural system associated with acetylcholine, which plays a role in attention and sensory precision.
Choral Speech: A fluency-enhancing condition in which a person who stutters speaks in unison with another speaker.
Confidence: A measure of precision in probabilistic inference, reflecting the certainty of predictions about sensory input or motor actions. Higher confidence corresponds to greater precision in prior expectations or policies.
Cortico-Basal Ganglia-Thalamo-Cortical Loop: A neural circuit that regulates motor control and speech, implicated in stuttering.
Covert Stuttering: Avoidance behaviors, word substitutions, or circumlocutions used to conceal stuttering.
Delayed Auditory Feedback (DAF): A technique where a speaker hears their own voice with a slight delay, which can either alleviate or induce stuttering depending on timing.
Distraction Effect: The reduction of stuttering when attention is directed away from speech, often through external stimuli or dual-task conditions.
Dopamine: A neurotransmitter involved in reward processing, motor control, and policy precision, often linked to stuttering due to its role in speech initiation and movement regulation.
Dystonia: A movement disorder characterized by involuntary muscle contractions, sometimes associated with stuttering-related tremors.
Epistemic Uncertainty: A lack of knowledge about a situation, leading to increased attention to incoming sensory information to resolve uncertainty.
Exteroception: The perception of external stimuli, such as auditory feedback during speech production.
Fluency Shaping: A speech therapy approach focused on modifying speech patterns to promote fluent speech.
Free Energy: A measure of uncertainty in the brain’s model of the world, which the brain actively seeks to minimize through perception and action.
Free Energy Principle: A theoretical framework, proposed by Karl Friston, stating that self-organizing biological systems minimize surprise (or free energy) by predicting and adapting to their environment.
Frequency-Altered Feedback (FAF): A method that changes the pitch of a speaker’s voice in real time, often used to modify stuttering behaviors.
Generative Model: A hierarchical neural model that predicts sensory input by integrating prior expectations with real-time sensory feedback.
Hierarchical Predictive Processing: A concept in active inference where higher-level brain areas generate predictions that are compared with lower-level sensory inputs to minimize prediction errors.
Homeostasis: The process of maintaining stable internal conditions in the body, contrasted with allostasis, which involves predictive regulation.
Law of Reversed Effort: A principle, proposed by Aldous Huxley, suggesting that the harder one tries to control a process (such as speech fluency), the more likely they are to experience failure due to excessive self-monitoring.
Interoception: The perception of internal bodily sensations, such as heartbeat, respiration, and muscle tension, which can influence speech production and stuttering.
Likelihood Function: In Bayesian inference, the probability of observing a particular sensory input given a specific state of the world.
Markov Decision Process (MDP): A mathematical framework for modeling decision-making where an agent transitions between states based on probabilistic policies.
Mistiming Hypothesis: A theory suggesting that stuttering arises from a phase shift in precision dynamics, causing a lack of sensory attenuation at the moment of syllable initiation.
Motor Planning: The process of preparing and sequencing movements, such as syllable production in speech.
Neural Oscillations: Rhythmic patterns of neural activity that coordinate information processing and motor control.
Neuromodulation: The regulation of neural activity by neurotransmitters like dopamine and acetylcholine, affecting sensory precision and motor control.
Norepinephrine: A neurotransmitter that influences attention, stress responses, and transition precision during uncertain situations.
Overt Stuttering: Observable speech disfluencies such as repetitions, prolongations, and blocks.
Perception-Action Cycle: A continuous feedback loop where sensory inputs inform motor actions, which in turn generate new sensory inputs.
Policy Precision: The confidence in selecting a specific sequence of actions or behaviors to achieve a goal with minimal uncertainty.
Predictive Coding: A neural mechanism where descending predictions from higher brain regions are compared with ascending sensory input to minimize prediction errors.
Prediction Error: The difference between expected and actual sensory input, which the brain attempts to minimize through adaptive action and perception.
Precision-Weighting: A process in active inference where the brain assigns confidence to prior expectations or sensory inputs to optimize prediction error minimization.
Principle of Least Effort: A linguistic principle, proposed by Georg Zipf, stating that human communication tends to minimize effort, influencing speech patterns and fluency.
Prior Precision: The confidence assigned to prior expectations based on past experiences, influencing how much weight they have in sensory processing.
Proprioception: The perception of the body’s position and movement, which contributes to motor control and speech fluency.
Sensory Attenuation: The suppression of self-generated sensory input to facilitate movement initiation, theorized to be impaired in individuals who stutter.
Sensory Gating: The brain’s ability to filter out unnecessary sensory information to facilitate smooth motor execution.
Sensory Precision: The confidence (inverse variance) assigned to sensory input, influencing how strongly it affects updating the generative model.
Sensorimotor Integration: The process by which sensory inputs (e.g., auditory, proprioceptive) are used to guide motor outputs in speech production.
Shadowed Speech: A fluency-inducing technique where a speaker immediately repeats words spoken by another person.
Fear Reduction Hypothesis: A theory, proposed by Joseph Sheehan, suggesting that repeated exposure to feared words or situations reduces anxiety and stuttering frequency.
Speech Motor Control: The neural and biomechanical processes that coordinate speech production.
Striatum: A subcortical structure within the basal ganglia involved in learning, motor planning, and speech fluency.
Syllable Sequencing: The coordination of successive syllables in speech production, often disrupted in stuttering.
Synaptopathy: A disorder affecting synaptic function, proposed as a neurobiological basis for stuttering due to disruptions in precision-weighting mechanisms.
Transition Precision: A measure of confidence in the ability to transition smoothly from one syllable or word to the next during speech.
Tremor: Involuntary muscle movements that can occur in speech articulators during moments of stuttering.
Uncertainty: A measure of unpredictability in sensory input or action selection, often linked to high prediction error and increased sensory precision.
Variational Free Energy: A computational quantity representing the difference between predicted and observed sensory input; minimizing it leads to more accurate predictions.
Volatility: The unpredictability of a communicative environment, which can increase uncertainty and disrupt fluent speech production.
Word Unpredictability Effect: The tendency for stuttering to be more likely on words that are unexpected or carry high information content.
